# Shift in GATA3 functions, and GATA3 mutations, control progression and clinical presentation in breast cancer

**DOI:** 10.1186/s13058-014-0464-0

**Published:** 2014-11-20

**Authors:** Helit Cohen, Rotem Ben-Hamo, Moriah Gidoni, Ilana Yitzhaki, Renana Kozol, Alona Zilberberg, Sol Efroni

**Affiliations:** The Mina and Everard Goodman Faculty of Life Science, Bar Ilan University, Ramat Gan, 52900 Israel

## Abstract

**Introduction:**

GATA binding protein 3 (GATA3) is a regulator of mammary luminal cell differentiation, and an estrogen receptor (ER) associated marker in breast cancer. Tumor suppressor functions of GATA3 have been demonstrated primarily in basal-like breast cancers. Here, we focused on its function in luminal breast cancer, where GATA3 is frequently mutated, and its levels are significantly elevated.

**Methods:**

GATA3 target genes were identified in normal- and luminal cancer- mammary cells by ChIP-seq, followed by examination of the effects of GATA3 expressions and mutations on tumorigenesis-associated genes and processes. Additionally, mutations and expression data of luminal breast cancer patients from The Cancer Genome Atlas were analyzed to characterize genetic signatures associated with GATA3 mutations.

**Results:**

We show that some GATA3 effects shift from tumor suppressing to tumor promoting during tumorigenesis, with deregulation of three genes, *BCL2*, *DACH1*, *THSD4*, representing major GATA3-controlled processes in cancer progression. In addition, we identify an altered activity of mutant GATA3, and distinct associated genetic signatures. These signatures depend on the functional domain mutated; and, for a specific subgroup, are shared with basal-like breast cancer patients, who are a clinical group with regard to considerations of mode of treatment.

**Conclusions:**

The GATA3 dependent mechanisms may call for special considerations for proper prognosis and treatment of patients.

**Electronic supplementary material:**

The online version of this article (doi:10.1186/s13058-014-0464-0) contains supplementary material, which is available to authorized users.

## Introduction

GATA binding protein 3 (GATA3) belongs to a family of tissue specific transcription factors regulating cell fate specification. Following binding to a consensus (A/T)GATA(A/G) sequence, GATA proteins transactivate their target genes either directly or through mechanisms involving long range chromatin remodeling and DNA looping [1],[2]. GATA3 is expressed in a variety of tissues, including the mammary gland, in which it is a key regulator of luminal cell lineage differentiation from mammary stem cells and maintenance of differentiated luminal epithelium within the mature gland [3],[4].

In breast cancer, GATA3 expression has been correlated with estrogen receptor positive (ER+/luminal) phenotypes [5]-[9], accounting for roughly two thirds of breast cancer cases [10], while loss of GATA3 expression is correlated with ER-, less differentiated, invasive breast cancer [7]. Accordingly, ectopic expression of GATA3 results in reversal of epithelial-mesenchymal transition (EMT), inhibition of dissemination and metastasis, and induction of differentiation in breast cancer cell lines and mouse models [11]-[13]. Thus, the activity of GATA3 in ER-, basal-like breast cancer (BLBC), has been largely attributed to negative regulation of genes associated with invasion and de-differentiation [14],[15]. These works and others [11],[16],[17] suggest that loss of GATA3 expression is involved in the aggressiveness of BLBC.

While the accumulating evidence points to tumor suppressor functions of GATA3, in luminal breast cancer GATA3 directly upregulates proto-oncogenes [1],[18]-[20], including the alpha subunit of ER (ERα) and co-regulates a large set of ERα target genes, suggesting that, contrary to the basal-like associated behavior, GATA3 may promote tumorigenesis in luminal breast cancer, either through ER dependent processes, or possibly, independent of ER.

Such studies highlighting a major role for GATA3 in breast cancer progression join sequencing studies demonstrating frequent somatic mutations in GATA3 in luminal breast cancer patients. These mutations are primarily within the DNA binding domain (DBD) of GATA3 and may modulate its activity [21]-[24].

These data combine to suggest that GATA3 is a master regulator in breast cancer via numerous molecular mechanisms, which may vary between BLBC and luminal breast cancer. A systematic identification of molecular pathways regulated by GATA3 would thus highlight and uncover major processes governing breast cancer development.

Here, we aimed to acquire a systemic view of the role played by GATA3 in luminal breast cancer progression. Hence, we identified its target genes in normal mammary and luminal breast cancer cells by chromatin immunoprecipitation coupled with massive parallel sequencing (ChIP-seq). We thus found a range of GATA3-associated mechanisms and a signature of genes which may be involved in breast cancer development. Strikingly, we found that a large proportion of GATA3 target sites are unique to either normal or cancer cells, and, furthermore, the regulatory effect of GATA3 is partially altered during cancer progression, shifting from a tumor suppressor in normal cells to a tumor promoting factor upon transformation. We identified further modulations to GATA3 function by altered activity of mutant GATA3, and associated genetic signatures in populations of luminal breast cancer patients. These signatures depend on the functional domain mutated and, for a specific subgroup, are shared with BLBC patients. These combine to define a distinct subpopulation, with possible clinical implications.

## Methods

### Analysis of gene expression data

Four independent datasets, available through the Gene Expression Omnibus (GEO [25]) database and containing gene expression and clinical data, were analyzed to identify biomarkers that could stratify breast cancer patients by clinical status: Lu *et al*. (GEO accession GSE5460) [26]; Wang *et al*. (GEO accession GSE2034) [27]; Ivshina *et al*. (GEO accession GSE4922) [28]; and Popovici *et al*. [29]. The Mann-Whitney U-test and Student’s t-test were performed on all gene expression measurements through ER status data to determine a gene’s stratification power. Genes that had significant *P*-values (<0.05) were compared in all four datasets to identify overlapping genes.

### ChIP-SEQ

ChiP was performed on 8 × 10^7^ cells, with 5 μg ChIP-grade anti GATA3 antibody (SC-268X, Santa Cruz Biotechnology, Dallas, TX, USA) per 1 × 10^7^ cells, using a ChIP assay kit (Millipore, Billerica, MA, USA), according to the manufacturer’s protocol. The eluted and input DNA were sequenced on SOLiD (Life Technologies–Applied Biosystems, Grand Island, NY, USA) -read sequences from CHIP-seq libraries were aligned to the human genome hg19 using Bowtie, allowing no more than two mismatches per read and using only the best reported valid alignments. To identify GATA3 binding sites, we used the Model-based Analysis for ChIP-Seq (MACS) to call peaks from the CHIP-seq data with default parameters. Extracted peaks showed at least three-fold enrichment relative to control input for each sample. For annotation, we used the ChIPpeakAnno, a Bioconductor R package, which includes functions to retrieve sequences around the peak and for finding the nearest site of interest, and the Gene Ontology (GO) for the associated pathways. Randomly selected binding sites were tested for sequence and verified by ChIP coupled with real time PCR, demonstrating the existence of a GATA3 binding site and binding affinities corresponding to relative peak intensities (data not shown).

### Cell lines

Human mammary epithelial cells (hMEC) were purchased from LONZA and grown on mammary epithelial growth medium (MEGM, LONZA, Boroline Road Allendale, NJ, USA); MDA-MB-231, MDA-MB-468, Hcc1143, T47D and MCF7 were purchased from ATCC (Manassas, VA, USA) and grown on high glucose Dulbecco’s modified medium containing L-glutamine (Invitrogen, Life Technologies, Grand Island, NY, USA) with addition of 10 mM 4-(2-hydroxyethyl)-1-piperazineethanesulfonic acid (HEPES), 1 mM sodium-pyruvate and 10% fetal calf serum. Sanger sequencing confirmed that wild type GATA3 was expressed in all of these cell lines.

### Plasmids

#### IL5/GFP

IL5 promoter fragment (-390 to +396) containing two GATA3 binding sites [30] in downstream of the GFP reporter gene.

#### wtGATA3; mutGATA3

cDNA of wild-type and mutant GATA3 alleles, respectively, of MCF7 cells, was isolated by PCR and cloned into pBabe/Zeo expression vector. Sanger-sequencing confirmed both the mutation and wild type sequences.

### Transient transfection assays

A total of 1 × 10^6^ cells/10 cm dish were transfected with 10 μg of DNA using JetPrime (POLYPLUS Transfection, Ill kirch, France) according to the manufacturer’s protocol. Total RNA was isolated 48 hours after the transfection, reverse-transcribed, and transcript levels quantified by real time PCR using ABI7900HT (Life Technologies-Applied Biosystems, Wilmington, MA, USA) with KAPA SYBR FAST ABI Prism 2X qPCR Master Mix (Kapa Biosystems). Relative expression levels were calculated as levels of tested genes in GATA3- over control (ctrl)- transfected cells each normalized to beta-actin. Primers used for real time PCR are detailed in Additional file 1: Table S2. Primers that were used for GATA3 detection react with both wild type and mutant GATA3.

### siRNA

Three different Stealth-RNAi siRNA duplex oligoribonucleotides for GATA3 and Stealth-RNAi siRNA negative control were purchased from Invitrogen. A total of 50 pmole of each duplex were transfected using JetPrime (POLYPLUS). Expression of GATA3 and target genes was tested 48 hours after transfection.

### Cancer progression model

hMEC cells were transformed as described in [31].

### Mammospheres assay

Cells were plated in ultra-low attachment tissue culture plates (Corning, Corning, NY, USA) as described by Dontu *et al*. [32], 48 hours after transfection. Mammospheres were measured and counted ten days after seeding under a NIKON TE2000 inverted microscope supplemented with a digital camera.

### Fluorescence activated cell sorting analysis

Cells were transfected as detailed above. Forty-eight hours after transfection, fluorescence was measured in 1 × 10^4^ GFP-positive cells using a Gallios flow cytometer (Beckman Coulter, Brea, CA, USA) and quantified using flowJo (TreeStar).

## Results

### Increased GATA3 levels characterize luminal breast cancer

**Figure 1:**
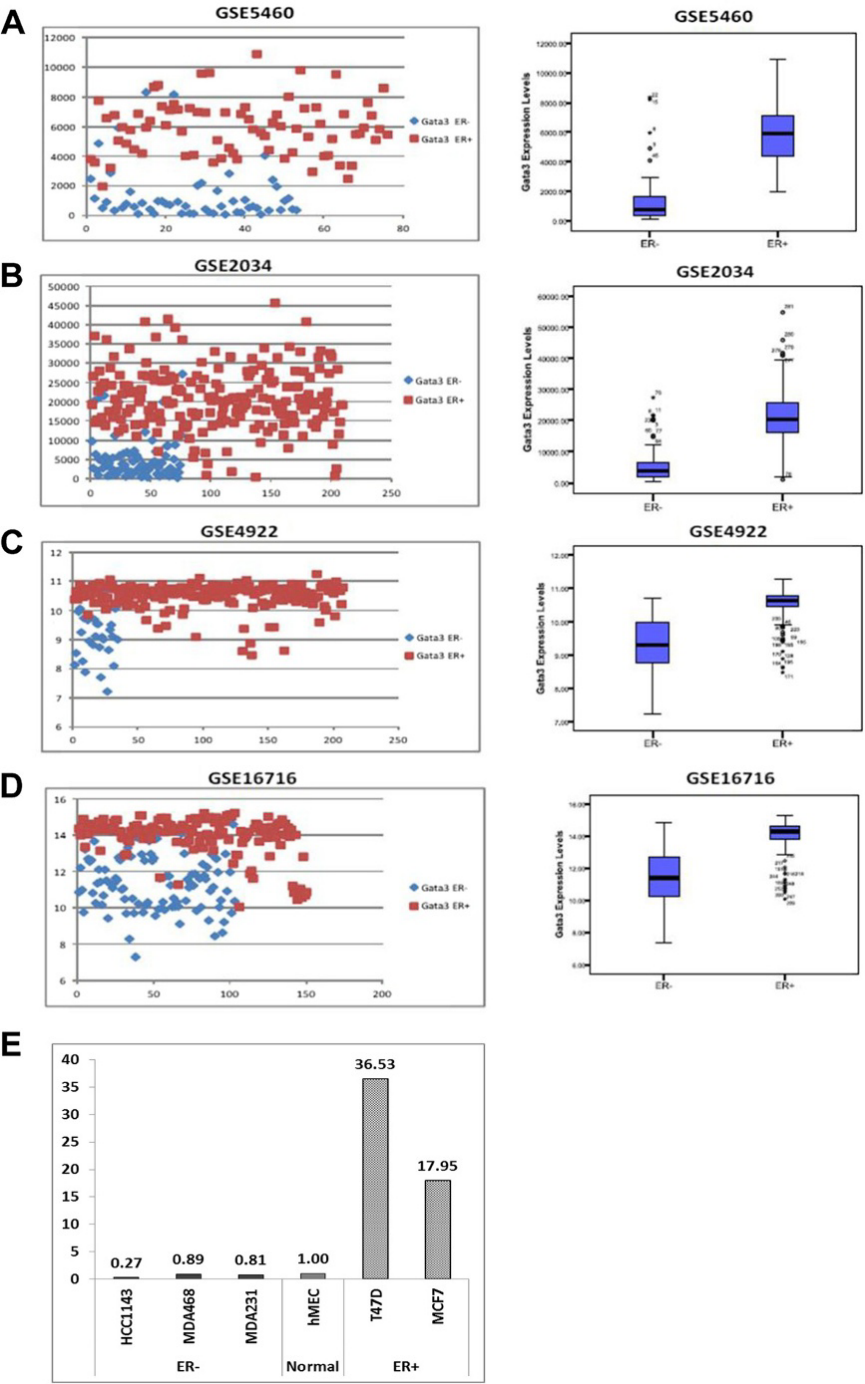
**GATA3 expression levels classify breast cancer patients in ER groups. (A-D)** GATA3 expression levels divide patients according to their ER status in multiple cohorts of patients. On each sub-figure, the left panel presents expression levels of GATA3 across the population of samples in each GEO dataset. ER- patients are tagged blue and ER+ patients are tagged red. The right panel presents the same data, in a bars and whiskers format. *P*-values for each dataset are: **(A)** 4.31E-27, **(B)** 3.49E-35, **(C)** 2.32E-17 and **(D)** 4.11E-40. **(E)** GATA3 expression levels are correlated with ER status in breast cancer lines. Relative GATA3 transcript levels in ER+ and ER- breast cancer lines are compared to normal mammary cells. GATA3 transcripts were detected by RT-qReal Time PCR and normalized to beta-actin. ER, estrogen receptor; GATA3, GATA binding protein 3.

Breast cancer patients are classified by molecular subtypes as a means of therapeutic decision making and prognosis [10]. In an effort to characterize molecular mechanisms associated with distinct breast cancer subtypes, we analyzed gene expression patterns in four publicly available cohorts of several hundred patients [26]-[29],[33],[34]. In agreement with published data [5]-[9], we found that the expression levels of GATA3 alone were sufficient to separate patients into ER positive and negative groups (Figure 1A-D). No other protein classified patients by clinically relevant groups at similar statistical significance in the roughly 1,000 patients tested (see Additional file 2: Table S1), adding to accumulating evidence that GATA3 plays an imperative role in the biology of breast cancer. We tested whether the variation in GATA3 levels reflects loss of expression in BLBC or increased expression in luminal breast cancer. To this end, we compared GATA3 transcript levels in the BLBC lines MDA-MB-468 (TNBC), MDA-MB-231 (Basal B) and Hcc1143 (TNBC) and in luminal breast cancer lines MCF7 and T47D to normal epithelial mammary cells. In accordance with the population effect, when compared to normal mammary epithelial cells, GATA3 transcript levels are elevated in the luminal breast cancer lines and decreased in BLBC lines (Figure 1E). Combined, these analyses indicate that GATA3 levels are altered in both basal-like (decreased levels) and luminal (increased levels) breast cancers. We postulated that various molecular pathways are altered following GATA3 overexpression in luminal breast cancer, which may promote cancer progression. The following experiments were designed to test this hypothesis.

### Genome-wide identification of GATA3 binding sites in normal mammary epithelial cells and luminal breast cancer cells

**Figure 2:**
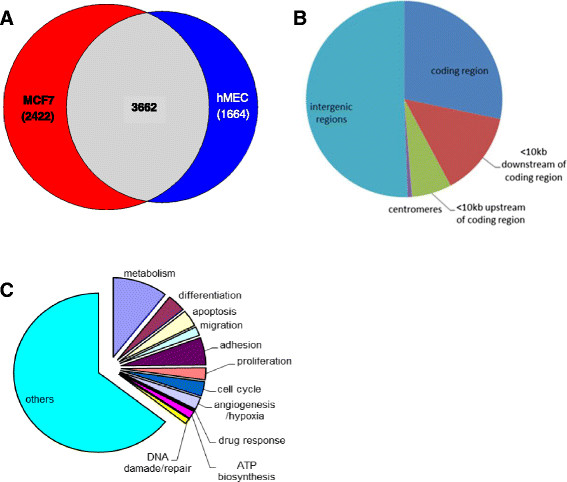
**Distribution of GATA3 target sequences. (A)** Venn diagram showing overlap of GATA3 target sites in MCF7 (red) and hMEC cells (blue); **(B)** Genomic distribution of GATA3 targets **(C)** Relative distribution of pathways associated with GATA3 target genes, as identified by ChIP-seq. ChIP, chromatin immunoprecipitation; GATA3, GATA binding protein 3; hMEC, human mammary epithelial cells.

As a first step in characterizing the involvement of GATA3 in luminal breast cancer, we identified its target genes in normal and cancer breast cells. We performed ChIP-seq of GATA3 in normal hMEC and MCF7 cells. hMEC are a mixed myoepithelial/luminal population, only the latter of which express GATA3 [35]; MCF7 is a luminal breast cancer line, which overexpresses high levels of GATA3, and thus is a convenient line to use for ChIP of the endogenous protein. Although these cells have one GATA3 allele with mutation within its DNA binding domain, the wild type allele is the one overexpressed and we postulated that its overexpression should overcome any effect that the mutant allele may have. Furthermore, the mutant allele has decreased affinity to the antibody we used, suggesting that DNA segments that are bound by the wild type allele will be primarily immunoprecipitated. Thus, these two lines were selected to compare and characterize GATA3 regulated genes in normal and cancer luminal mammary cells. For each cell type, input and GATA3 bound DNA from two independent experiments were sequenced on a SOLiD sequencer. At a *P*-value of 0.001, 5,266 binding sites in hMEC and 6,084 in MCF7 cells were at least three-fold enriched above the respective input samples. Thirty percent were cell-specific (Figure 2A), pointing to altered targets recognition by GATA3 following tumorigenesis.

In both samples, 46% of GATA3 binding sites were inside or within 10 kbp of coding sequences (Figure 2B). Of these, among GO annotated genes [36], large groups are associated with cellular/organ differentiation, adhesion and migration, in accordance with published data [4],[12],[13] (Figure 2C). Other groups of GATA3-regulated genes were found associated with processes which may be involved in cancer development and progression, including regulation of metabolic processes, proliferation, cell cycle, apoptosis, angiogenesis and hypoxia. These processes were parallel in hMEC and MCF7 (data not shown). However, since the majority of the genes are not annotated, this observation cannot be conclusive.

**Figure 3:**
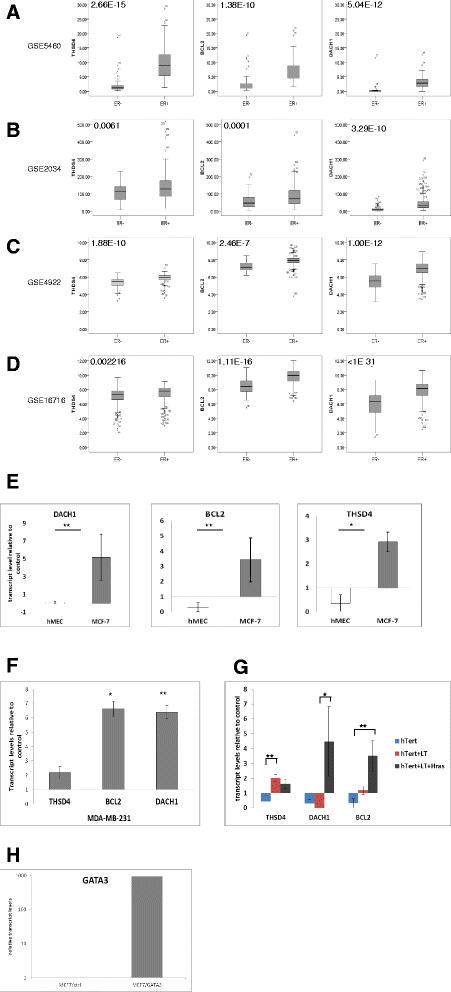
**GATA3 controls luminal breast cancer predominantly through differential regulation of DACH1, THSD4 and BCL2 genes. (A-D)** Expression levels of GATA3 target genes stratify patients by ER status in the same cohorts of patients as used in Figure 1. Each sub-figure presents two patient groups. *P*-values are presented at the upper-left corner of each graph. **(E)** GATA3 has opposite effects on THSD4, BCL2 and DACH1 in normal cells and cancer cells, respectively. Expression levels of specified genes after transfections of GATA3 relative to vector into indicated cells are presented. **(F)** Upregulation of THSD4, BCL2 and DACH1 by GATA3 is independent of ER expression: relative levels of the indicated genes after transfection of GATA3 or empty vector into MDA-MB-231 cells are presented. **(G)** Deregulation of THSD4, BCL2 and DACH1 during tumorigenesis: hMEC were transformed by sequential introduction of the three genetic elements detailed in the figure. Effect of GATA3 expression at each step was assessed as above. **(H)** Representative expression of GATA3 following transfection. Results shown in figures E-G are average ± standard error of three to five independent experiments. (*) *P*-value <0.1; (**) *P*-value <0.05. ER, estrogen receptor; GATA3, GATA binding protein 3; hMEC, human mammary epithelial cells.

To explore major GATA3-regulated genes which may be involved in breast cancer, we correlated expression levels of the GATA3 target genes with clinical data available from breast cancer patients [26]-[29],[33],[34]. Three genes (described in details in Table 1): BCL2 – an anti-apoptotic protein [37]; DACH1 - inhibitor of ER signal transduction and apoptosis and regulator of cell cycle progression [38]-[40]; and THSD4, which is possibly associated with extra cellular matrix assembly [41], divided breast cancer patients according to ER status (Figure 3A-D), suggesting that these three genes and their related functions may participate in major GATA3-controlled breast cancer pathways.

**Table 1:** **Details of GATA3 target genes tested in this work**

Gene	Abbreviation	Function	Reference
B-cell CLL/lymphoma 2	BCL2	Anti-apoptotic	[37]
Dachshund1	DACH1	• Anti-apoptotic	[38]-[40],[42],[43]
		• Inhibitor of estrogen signaling	
		• Cell cycle progression	
		• Inhibitor of breast cancer stem cells	
		• Inhibitor of tumor growth and metastasis	
Thrombospondin, type I domain containing 4	THSD4	May promote matrix assembly	[41]
Solute carrier organic anion transporter family, member 5A1	SLCO5A1	Nutrients (possibly hormones) uptake	[44]
Growth regulation by estrogen in breast cancer 1	GREB1	Estrogen induced breast cancer cells growth/ proliferation	[45]
Brain-enriched guanylate kinase-associated homolog	BEGAIN	Chromosome segregation	[46]
Centrosomal protein 70 kDa	CEP70	• Mitotic spindle assembly	[47],[48]
		• Angiogenesis	
Rho-associated, coiled-coil containing protein kinase	ROCK1	Serine/threonine kinase	[49]-[52]
		• Focal adhesion	
		• Cancer invasion/ metastasis	
		• Angiogenesis	
		• Apoptotic membrane blebbing	
SUMO1/sentrin specific peptidase 5	SENP5	SUMO specific protease	[53],[54]
		• Cell division	
		• Proliferation	
Kinesin family member 16B	KIF16B	Endosome transport and receptor recycling and degradation	[55],[56]
		• FGF/EGF signal transduction	
Potassium channel tetramerisation domain containing 2	KCTD2	Ion transport	[57]
		• Upregulated in liver metastasis	
ATP-dependent DNA helicase homolog	HFM1	• Putative DNA helicase	[58]
		• Susceptibility to soft-tissue sarcoma	
		• Extensive tumor aneuploidy	
Bone morphogenetic protein 2	BMP2	• TGF-β receptor antagonist	[59]-[61]
		• Cancer invasion/migration	
		• Cancer proliferation	
		• Tumor angiogenesis	
		• Hormone independence	
v-erb-a erythroblastic leukemia viral oncogene homolog 4	ERBB4 (HER4)	• EGF receptor	[62],[63]
		• Mammary gland differentiation	
		• Apoptosis	
		• Cell cycle and proliferation arrest in breast cancer

### Alteration in GATA3 regulatory effect upon transformation

Of the three ER associated GATA3 targets identified above, BCL2 has the best characterized tumor-promoting activity, while DACH1 has been reported to function as both tumor promoter [42] and tumor suppressor [38]. To test the effects of GATA3 on tumorigenesis, we, therefore, tested its effect on these genes in normal and luminal breast cancer cells. Overexpression of GATA3 in normal hMEC cells resulted in downregulation of BCL2, DACH1 and THSD4, supporting a tumor suppressor function for GATA3 under normal conditions. However, in the luminal breast cancer lines MCF7 and T47D, these genes were induced by GATA3 overexpression (Figure 3E, H and Additional file 3: Figure S1), suggesting that upon transformation, GATA3 may change its function to support cancer progression. This effect was not limited to the ER expressing lines, as a parallel effect was demonstrated in the ER- line MDA-MB-231 (Figure 3F). An alternative interpretation could be that the difference in activity resulted from a difference in the lineage of hMEC compared to the breast cancer cells tested. We thus tested the effect of GATA3 in a stepwise transformation model, in which hMEC are transformed by sequential introduction of genes encoding the telomerase catalytic subunit hTert, SV40 large-T antigen (LT) and the oncoprotein H-Ras [31].

In hTert- immortalized cells, exogenous expression of GATA3 resulted in downregulation of BCL2, THSD4 and DACH1 levels, similar to hMEC. Upon subsequent transformation, however, a shift concomitant with our observations in luminal breast cancer cells occurred, namely, GATA3 upregulates expression of these genes (Figure 3G).

The shift in regulatory activity demonstrates opposing tumor suppressor and tumor promoting associated effects of GATA3 in normal and breast cancer cells, respectively. Furthermore, deregulation of BCL2, DACH1 and THSD4 may represent key events accompanying GATA3-mediated transformation of normal cells into breast cancer.

### Genes associated with various cellular processes are deregulated following altered GATA3 function

Cancer is driven by an accumulation of alterations in processes dictating the normal functions of a cell. While the three genes we identified above may have a central role in breast cancer progression, other GATA3-regulated processes may be altered upon transformation.

**Figure 4:**
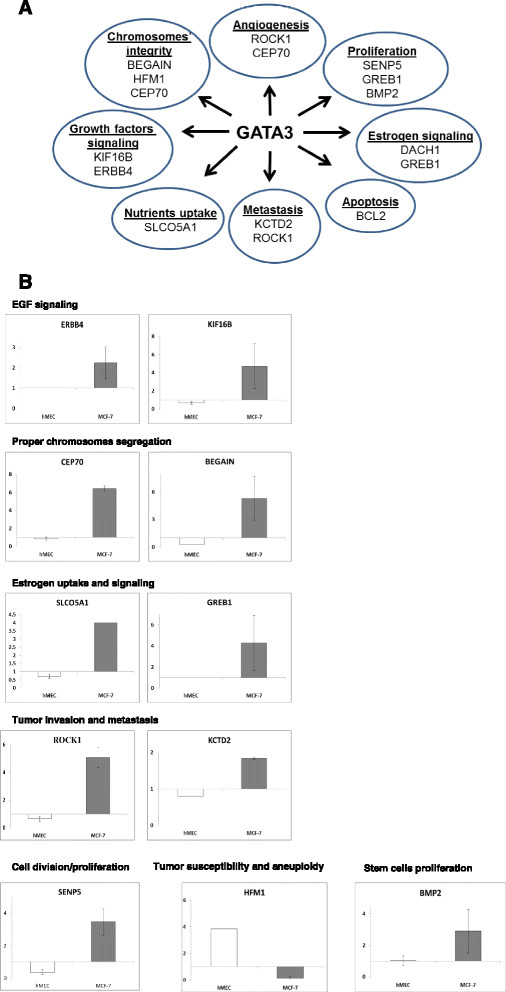
**Response of target genes to GATA3 depends on the cell’s condition. (A)** Representative GATA3-controlled processes and associated genes. **(B)** Relative expression levels of GATA3 target genes after transfection of GATA3 or empty vector into the indicated cells. Results shown are average ± standard error of two to three independent experiments. GATA3, GATA binding protein 3.

To study GATA3-associated mechanisms involved in normal cell functions and luminal breast cancer, we selected genes (from those identified by ChIP-seq), representing different molecular processes according to the GO browser (Figure 4A; and Table 1), and their response to GATA3 was examined in hMEC and in MCF7 cells.

In accordance with our former observations, the response to GATA3 was cell-context dependent (Figure 4B). Namely, in MCF7 cells, all of the genes tested were upregulated in response to overexpression of GATA3 (Figure 4H), with the exception of HFM1, which was downregulated. These results were verified in MCF7 and T47D cells for representative genes by transfection of three different GATA3 siRNA (see Additional file 4: Figure S2). However, in normal mammary cells, again with the exception of HFM1, the genes tested were either downregulated or did not respond to GATA3 (Figure 4B).

Combined, the differences in GATA3 target genes and responses indicate that alterations in GATA3 regulatory effect depend on the cellular settings (that is, normal *versus* cancer cells), possibly reflecting other transcription factors expressed in the cell.

### GATA3 induces an increase in proliferating stem cell populations of cancer, but not healthy, mammary cells

The altered regulatory effect of GATA3 in breast cancer cells predicts that a distinct phenotype should result in normal and cancer cells following GATA3 expression. In normal luminal mammary cells, GATA3 is associated with differentiation of progenitor cells. Accordingly, a group of genes that we identified by ChIP-seq and that responded differentially to GATA3 (for example, BMP2, ERBB4 and KIF16B [26],[64]) are linked to proliferation and maintenance of normal or cancer stem cells. We therefore tested the effect of GATA3 on populations of normal or cancer stem cells, as follows: 48 hours following transfection of GATA3 or control vector, hMEC or MCF7 cells were seeded in ultra-low attachment plates according to a published protocol [32], and floating spheres, representing progenitor [32] or tumor stem cells (tumor initiating cells (TICs)) [65], respectively, were counted after 10 days.

**Figure 5:**
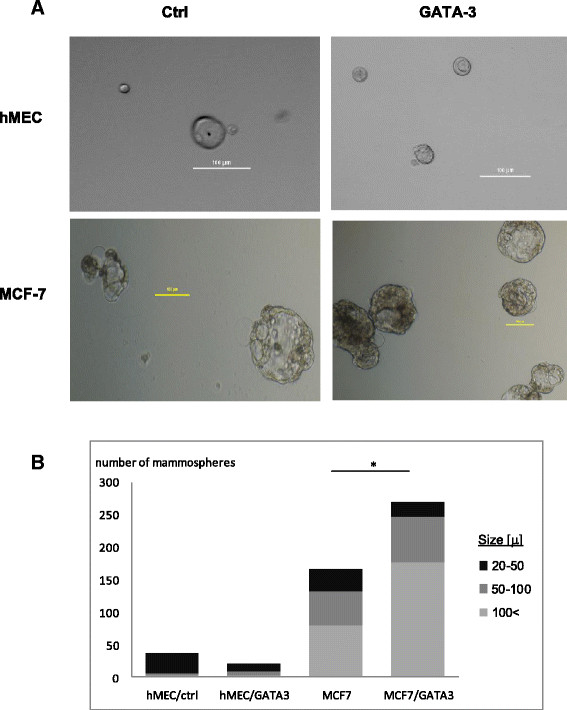
**GATA3 induces populations of proliferating mammospheres in breast cancer, but not normal, cells. (A)** Representative mammospheres generated following control or GATA3 transfection. A 100 μ bar is shown at each photo. **(B)** Mammospheres count. Average counts of two independent experiments for each cell type are shown. (*) T-test *P*-value <0.1. GATA3, GATA binding protein 3.

In accordance with published data [66], expression of GATA3 resulted in a decrease in the numbers and sizes of the mammosphere population isolated from hMEC (Figure 5A-B). Conversely, in MCF7 cells, an increase in floating sphere populations, and specifically those that were above 100 μ in size, was observed in the GATA3 transfected culture. These results indicate that GATA3 plays a role in the proliferation of cancer stem cells and, in contrast, the decrease of normal stem cell populations.

### Mutation of GATA3 leads to altered expression of target genes

While, so far, we have focused on the effects of GATA3 overexpression in cancer cells, an additional alteration to GATA3 functional activity may be associated with mutations found in a population of luminal breast cancer patients. In MCF7 cells a G insertion at position 1566 resulting in frameshift at D366 and truncation of the DBD has been characterized [21]-[24]. This set of mutations may impair GATA3 regulatory activity and lead to distinct tumor characteristics of the population which has mutated GATA3 allele. The next experiments were designed to explore the possibility of altered activity of mutant GATA3, using the mutant (mut) or wild type (wt) alleles isolated from MCF7 cells.

**Figure 6:**
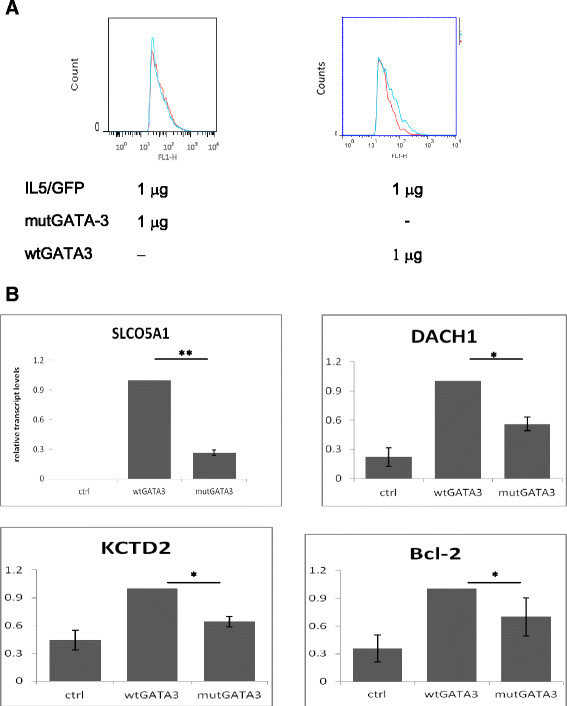
**Altered effect of mutant GATA3 in breast cancer leads to a distinct expression pattern of GATA3 target genes. (A)** mutGATA3 fails to activate GATA3 dependent promoter. GFP construct driven by GATA3 responsive promoter was cotransfected with the indicated constructs into HeLa cells and the fluorescence levels measured after 48 hours. Representative results of three independent experiments are shown. In each section, the red curve represents GFP transfected cells; the blue curve represents GFP with mut- or wtGATA3, as indicated. **(B)** mutGATA3 has an altered effect in breast cancer cells. Relative transcript levels of GATA3 target genes after transfection of wtGATA-3 or mutGATA-3 into MCF7 cells. Results shown are average ± standard error of three to four independent experiments. GATA3, GATA binding protein 3; mut, mutant; wt, wild type. (*) *P*-value <0.1 (**) *P*-value <0.005.

We first tested whether the mutant GATA3 from MCF7 cells (mutGATA3) was capable of activating a target gene. To this end, an IL5/GFP construct was cotransfected with wtGATA3 or mutGATA3 into HeLa cells, and GFP levels were measured 48 hours post transfection. Expression levels of wtGATA3 and mutGATA3 genes were comparable, as verified by qRT-PCR (data not shown). As expected, wtGATA3 activated the expression of IL5/GFP. However, mutGATA3 failed to activate IL5/GFP expression indicating that the DBD truncation associated with luminal breast cancer diminishes GATA3 activity (Figure 6A).

The inability of mutant GATA3 to activate the IL5 promoter raises the possibility that it might have a distinct effect on a subset of promoters, in which GATA3 is necessary to recruit other transcription factors.

To test this, we overexpressed the mutGATA3 allele in MCF7 cells and compared the levels of GATA3 regulated genes to wtGATA3 transfected cells.

In agreement with our hypothesis, we found that mutGATA3 had weaker activity or failed to regulate GATA3 target genes (Figure 6B), concomitant with altered activity. These observations suggest that mutant GATA3 protein may lead to disparate cancer associated mechanisms.

### Distinct molecular signatures in luminal breast cancer patients harboring GATA3 mutations depend on the functional domain mutated

The altered effect of mutant GATA3, in conjunction with the central role for GATA3 in the biology of breast cancer, predicts that mutation within GATA3 sequence may lead to altered gene expression patterns in patients, with possible clinical implications. To characterize a mutant GATA3-associated signature, we analyzed mutation data of luminal breast cancer patients from The Cancer Genome Atlas [67],[68]. Of these patients, 14.7% (47/319 patients) had heterozygous mutation for GATA3 (henceforth termed wt/mutGATA3). These mutations were distributed within intron 4 (splice site mutations; twelve patients) and intron 5 (splice site mutations; four patients), and within exon 3 (missense mutations; three patients), exon 5 (frameshift mutations; eight patients) and exon 6 (frameshift mutations; twenty patients). Total levels of GATA3 expression in the wt/mutGATA3 patients were comparable to those of the wtGATA3 population (data not shown).

**Figure 7:**
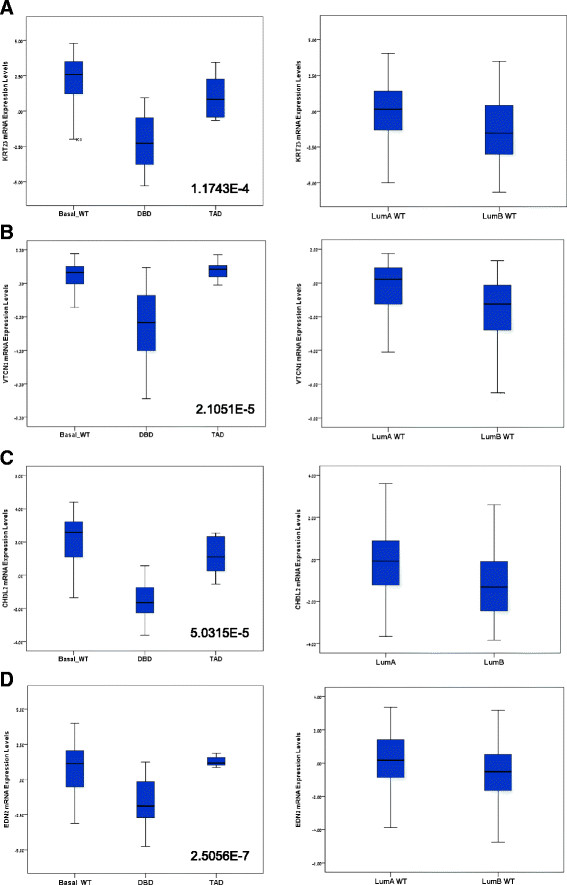
**Distinct genetic signatures of luminal breast cancer patients are associated with specific mutations of GATA3. (A**-**D)**: Mean and distribution of expression levels of indicated genes are shown. The basal WT group are BLBC patients with no GATA3 mutations; DBD and TAD are luminal breast cancer patients heterozygous for GATA3 mutations within the DBD (exons 5, 6) or TAD (intron 4, 5 or exon 3), respectively. T-test *P*-values of the differences between the DBD and TAD groups are presented at the right bottom corner of each graph. BLBC, basal-like breast cancer; DBD, DNA binding domain; GATA3, GATA binding protein 3; TAD, transactivation domain; WT, wild type.

Analyzed as a single group, the wt/mutGATA3 patients do not present a distinct molecular characteristic. We divided the patients into groups, according to the site of the mutations within the GATA3 sequence. The groups that generated clear signatures corresponded to the functional domains [69] mutated: patients bearing a mutation within the DBD (exons 5,6) and patients bearing mutations within the transactivation domain (TAD, intron 4,5, exon 3). These groups produced a genetic signature comprised of four genes: *CHI3L2*, *KRT23*, *VTCN1* and *EDN2*, according to which the mutant TAD patients were clustered with BLBC patients and mutant DBD patients formed an altogether discrete group (Figure 7A-D, left panels). Within the entire population of luminal breast cancer patients, these genes did not divide the patients by subtypes (Figure 7A-D; right panels).

These results demonstrate that distinct breast cancer subtypes are associated with wt/mutGATA3 populations within luminal breast cancer patients, and may suggest that these patients share some of the GATA3-dependent mechanisms of BLBC patients, who are a clinical group with regard to considerations of mode of treatment.

## Discussion

Breast cancer is, in fact, a collection of alterations in genomic, genetic and expression profiles, leading to a group of breast diseases [70]. The heterogeneity of the disease(s) creates a challenge in identifying an exclusive driving force and, consequently, early diagnosis, decision on mode of treatment and prognosis [71]. Clinically implemented signatures today use different biomarkers, such as combined expression and genomic variation differences, to classify patients in clinical groups as a means of decision on mode of treatment. The unique ability of GATA3 as a sole biomarker, that is, not as a part of a genomic signature, to classify breast cancer patients in clinical groups is, thus, uncommon in the context of breast cancer, and suggests that GATA3 is a major force behind processes standing at the core of the disease. While much attention has been given to tumor suppressor functions of GATA3 [4],[12],[13],[39],[72],[73], lately it was reported to mediate ER binding to the genome, thus supporting growth of hormone-driven cancers [1],[20]. Our findings connect these different functions by suggesting a cellular-context dependent equilibrium between tumor suppressor and tumor promoting effects. Based on altered regulatory activity of GATA3 upon tumorigenesis, we can postulate that in normal cells GATA3 is associated with tumor suppression activities (for example, differentiation, proper apoptotic processes, reduced proliferation). Conversely, in luminal breast cancer, GATA3 supports aspects of cancer progression through altered signal transduction of hormones and growth factors; increased proliferation and decreased apoptosis; and simultaneously, signaling prognosis associated with GATA3 expression [7],[74] may be a result of other processes, including upregulation of genes regulating chromosome integrity (BEGAIN, CEP70, HFM1).

Within the host of GATA3 target genes we uncovered, a signature of three genes (*BCL2*, *DACH1* and *THSD4*) separates patients into ER groups, underlining deregulation of apoptotic signaling (negatively regulated by *BCL2* and *DACH1*), ER signaling and cell cycle control (regulated by *DACH1* [38]-[40],[42],[43]) as possible major processes early in luminal breast cancer development. The function of *THSD4*, currently unknown, may expose additional pathways involved in luminal breast cancer progression. In BLBC, other GATA3-controlled cellular pathways, or lack of downstream ER signaling, may compensate for upregulation of BCL2, THSD4 and DACH1 by GATA3, resulting in a tumor suppressor function.

Thus, a model for a GATA3-dependent mechanism controlling transformation involves initial alterations in the milieu of transcription factors expressed in a cell, followed by loss of control on cell cycle progression, apoptosis and ER signaling resulting from deregulation of BCL2, DACH1 and/or THSD4 by GATA3. These changes, in turn, permit further acquisition of tumorigenic-associated signals leading eventually to cancer. This model suggests that GATA3 activity precedes, or, alternatively, circumvents the need for ER signaling in early tumorigenesis, in agreement with supportive evidence [1], thus placing GATA3 at the core of luminal breast cancer.

The effect of GATA3 results, however, not only from direct transactivation of targets. GATA proteins have an established function in locus control regions, directing tissue- and developmental-specific expression patterns of distal regions [75],[76]. Indeed, GATA3 binding sites as identified by ChIP-seq are predominantly enriched in intergenic regions. Accordingly, an altered binding pattern in cancer cells would influence the cell’s transcription program through chromatin organization, and sequentially transcription, of extensive and distal genomic regions.

The dual activities of GATA3 are exemplified by its effect on normal/cancer stem cell populations. In normal cells, GATA3 expression reduced the population of progenitor cells, in agreement with a role in differentiation of these cells into mature luminal cells [4],[66],[72]. Conversely, in luminal cancer cells, GATA3 expression led to proliferation of TICs. Since, in luminal breast cancer models, GATA3 induces differentiation [12],[66], we postulate that our observations reflect proliferation of an existing TIC population rather than active de-differentiation. This is consistent with findings in a mouse model, which showed that exogenous expression of GATA3 leads to larger, but more differentiated, tumors, relative to control tumors [12]. Thus, GATA3 acts to prevent tumorigenesis in normal cells by reducing populations of transformation susceptible progenitor cells, while in luminal breast cancer it induces both differentiation, and, as a tumor-supporting factor, proliferation of therapy-resistant [77], EMT-associated [78], TICs. Further functional tests will be needed to fully characterize the equilibrium between tumor supporting- and suppressing-functions of GATA3.

GATA3 is one of the frequently mutated genes in breast cancer [21],[22],[79], predominantly in the luminal subtype [79]. Distinct GATA3 regions necessary for chromatin remodeling and direct transactivation [80] predict that mutations of different domains may affect different sets of genes. Accordingly, mutations and expression analyses demonstrated distinct molecular signatures associated with mutations of specific functional domains. Clustering of patients bearing TAD mutations with BLBC patients may result from reduced activity on target genes [81] mimicking the phenotype of GATA3-low breast cancer, while an altogether divergent and discrete signature characterizes patients with DBD mutations. Further experiments are needed to fully characterize the molecular basis and possible clinical outcome associated with the different mutations. However, these molecular signatures suggest that molecular typing and, subsequently, prognosis and treatment considerations for breast cancer patients may require incorporation of specific GATA3 mutations.

## Conclusions

In summary, a critical role is demonstrated for GATA3 within the networks that govern breast cancer progression. Changes in genomic targets and regulatory activity may control tumor-associated mechanisms. Considerations of mutational modifications demonstrate how a GATA3 positive cancer may actually produce a distinct clinical phenotype. Our findings combine to expose possible molecular mechanisms associated with breast cancer progression and suggest that typing of patients according to their GATA3 behavior may add a layer in therapeutic considerations.

## Additional files

## Electronic supplementary material

Additional file 1: Table S2.: Primers used in real time PCR. (DOC 40 KB)

Additional file 2: Table S1.: T-test *P*-values for genes dividing breast cancer patients groups by ER status at the highest statistical significance. (DOC 31 KB)

Additional file 3: Figure S1.: GATA3 induces Bcl2, DACH1 and THSD4 in luminal breast cancer lines. Relative expression levels of specified genes were measured 48 hours following siRNA transfections relative to Ctrl siRNA transfected cells, both normalized to beta-actin. Results of three pooled GATA3 siRNAs are shown. (PDF 102 KB)

Additional file 4: Figure S2.: Silencing of GATA3 is followed by downregulation of tested genes in luminal breast cancer cells. Three pooled siRNA were used to silence GATA3 in MCF7 or T47D lines (a). Relative expression levels of tested genes were measured in MCF7 (b) and T47D (c) cells transfected with GATA3 siRNA relative to control transfected cells, both normalized to beta-actin. Results are average ± standard error of three to five independent experiments. *T-Test *P*-values <0.1; **T-Test *P*-values <0.05; ***T-Test *P*-values <0.01. (PDF 272 KB)

Below are the links to the authors’ original submitted files for images.

Authors’ original file for figure 1

Authors’ original file for figure 2

Authors’ original file for figure 3

Authors’ original file for figure 4

Authors’ original file for figure 5

Authors’ original file for figure 6

Authors’ original file for figure 7

## Abbreviations

BLBC: basal-like breast cancer
ChIP: chromatin immunoprecipitation
DBD: DNA binding domain
EMT: epithelial mesenchymal transition
ER: estrogen receptor
GATA3: GATA binding protein 3
GFP: green fluorescent protein
GO: gene ontology
hMEC: human mammary epithelial cells
siRNA: small interfering RNA
TAD: trans-activation domain
TIC: tumor initiating cells
