# Supplementary material for: Shift in GATA3 functions, and GATA3 mutations, control progression and clinical presentation in breast cancer
Source: Breast Cancer Res. 2014 Nov 20;16:464. doi: 10.1186/s13058-014-0464-0 (PMC4303202; doi:10.1186/s13058-014-0464-0)
Supplement: Supplementary file 1 — Additional file 1: Table S2.: Primers used in real time PCR. (DOC 40 KB) [file 13058_2014_464_MOESM1_ESM.doc]

**Additional file 1: Table S2: Primers used in real time PCR**

| **Reverse primer** | **Forward primer** | **Gene** |
| --- | --- | --- |
| 5' GAAGTCCTCCAGTGAGTCAT 3' | 5' CTCTACTACAAGCTTCACAAT 3' | **GATA3** |
| 5' GCCGGTTCAGGTACTCAGT 3' | 5' GAGGATTGTGGCCTTCTTTG 3' | **BCL2** |
| 5' TAGAACTTGCGTTGGTGCAG 3' | 5' GGTGTGCAATGTGGAACAAG 3' | **DACH1** |
| 5' ACTTCACATCACGGGTCCTC 3' | 5' GGAGAAACCTGAGACCACCA 3' | **THSD4** |
| 5' GACCCCGAACCGTCCCTAGAGG 3' | 5' TTCCGGGACTGGAGACTGCAGC 3' | **SLCO5A1** |
| 5' GTGCCAGCTGGACCAGGTAGT 3' | 5' TACGAGCAGTACGGCGCCTCT 3' | **GREB1** |
| 5' CCTTGGCTTCTAGCAGATGG 3' | 5' CTACATGGCACTGCAGAGGA 3' | **BEGAIN** |
| 5' TTCTTCTTGCTCCGTTCGTT 3' | 5' CTAAGTAGGGCTTGCCACCA 3' | **CEP70** |
| 5' GCTACAGTGTCTCGGAGCGT 3' | 5' GACAGGGAAGTGAGGTTAGG 3' | **ROCK1** |
| 5' GCGAGGCACTTGCAGTACTGGAG 3' | 5' GGGTTGGCAGACTGCTGTTACGA 3' | **SENP5** |
| 5' GAATCCCTGTAAGGCACGAA 3' | 5' GTGACTCTGGGGAACGTCAT 3' | **KIF16B** |
| 5' CGTCCCAGACCTGGGGCAGA 3' | 5' TGCGTGGCAGTTTGGGGCTG 3' | **KCTD2** |
| 5' TTTGCCGGAATTTCTGTGACA 3' | 5' GAGAAGGCATGTTCAAAGCAC 3' | **HFM1** |
| 5' AGCCACAATCCAGTCATTCC 3' | 5' TCAAGCCAAACACAAACAGC 3' | **BMP2** |
| 5' AGGAGGCCATCTTCCATCTT 3' | 5' GACCAATGTCTGTCGTGTCG 3' | **ERBB4** |
