# Supplementary material for: Shift in GATA3 functions, and GATA3 mutations, control progression and clinical presentation in breast cancer
Source: Breast Cancer Res. 2014 Nov 20;16:464. doi: 10.1186/s13058-014-0464-0 (PMC4303202; doi:10.1186/s13058-014-0464-0)
Supplement: Supplementary file 2 — Additional file 2: Table S1.: T-test P-values for genes dividing breast cancer patients groups by ER status at the highest statistical significance. (DOC 31 KB) [file 13058_2014_464_MOESM2_ESM.doc]

| **GSE16716** | **GSE4922** | **GSE2034** | **GSE5460** | **Gene** |
| --- | --- | --- | --- | --- |
| **4.11E-40** | **2.32E-17** | **3.49E-35** | **4.31E-27** | **GATA3** |
| 2.59E-36 | 7.95E-15 | 8.26E-21 | 1.35E-22 | TBC1D9 |
| 3.91E-36 | 4.28E-15 | 7.69E-10 | 9.73E-25 | CA12 |
| 1.32E-29 | 4.12E-07 | 5.64E-17 | 4.22E-17 | VAV3 |

**Additional file 2: Table S1: T-test P-values for genes dividing breast cancer patients groups by ER status at the highest statistical significance**
