# Supplementary material for: Shift in GATA3 functions, and GATA3 mutations, control progression and clinical presentation in breast cancer
Source: Breast Cancer Res. 2014 Nov 20;16:464. doi: 10.1186/s13058-014-0464-0 (PMC4303202; doi:10.1186/s13058-014-0464-0)

Figure S1: GATA3 induces Bcl2, DACH1 and THSD4 in luminal breast cancer lines

Relative expression levels of specified genes were measured 48 hours following siRNA transfections relative to Ctrl siRNA transfected cells, both normalized to beta-actin. Results of three pooled GATA3 siRNAs are shown.

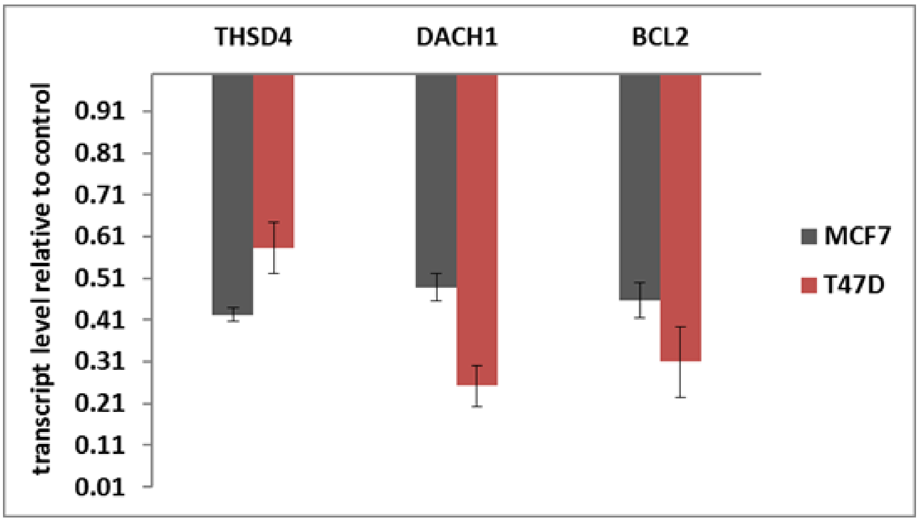

Supplement: Supplementary file 3 — Additional file 3: Figure S1.: GATA3 induces Bcl2, DACH1 and THSD4 in luminal breast cancer lines. Relative expression levels of specified genes were measured 48 hours following siRNA transfections relative to Ctrl siRNA transfected cells, both normalized to beta-actin. Results of three pooled GATA3 siRNAs are shown. (PDF 102 KB) [file 13058_2014_464_MOESM3_ESM.pdf]
