# Supplementary material for: Shift in GATA3 functions, and GATA3 mutations, control progression and clinical presentation in breast cancer
Source: Breast Cancer Res. 2014 Nov 20;16:464. doi: 10.1186/s13058-014-0464-0 (PMC4303202; doi:10.1186/s13058-014-0464-0)

Figure S2: Silencing of GATA3 is followed by downregulation of tested genes in luminal breast cancer cells.

Three pooled siRNA were used to silence GATA3 in MCF7 or T47D lines (a). Relative expression levels of tested genes were measured in MCF7(b) and T47D (c) cells transfected with GATA3 siRNA relative to control transfected cells, both normalized to beta-actin. Results are average±standard error of three to five independent experiments. * T-Test P-values < 0.1; ** T-Test P-values < 0.05; *** T-Test P-values < 0.01

a

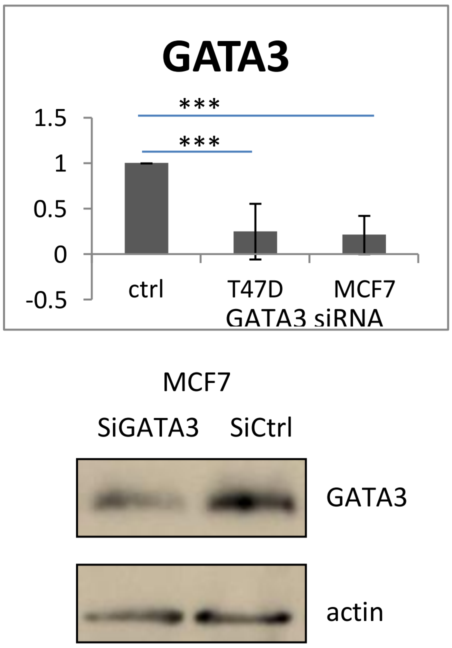

b

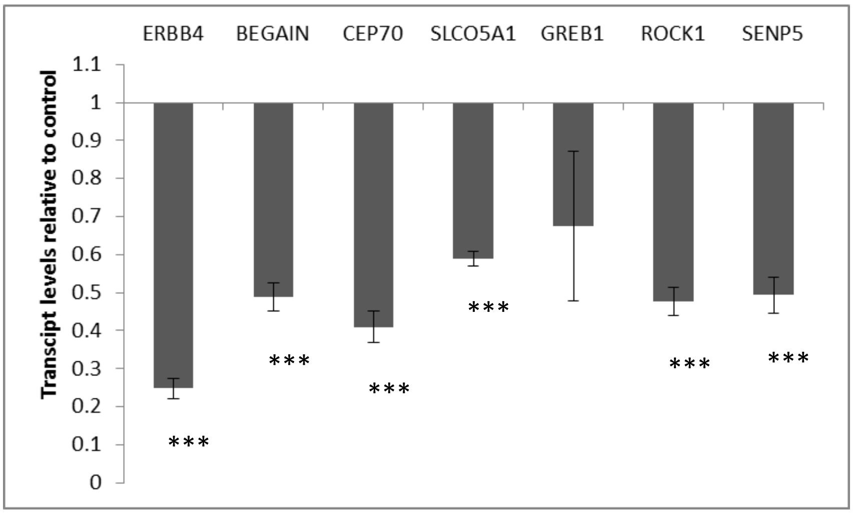

c

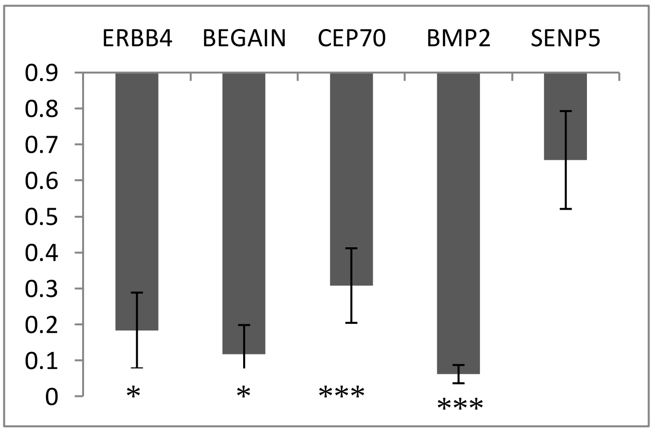

Supplement: Supplementary file 4 — Additional file 4: Figure S2.: Silencing of GATA3 is followed by downregulation of tested genes in luminal breast cancer cells. Three pooled siRNA were used to silence GATA3 in MCF7 or T47D lines (a). Relative expression levels of tested genes were measured in MCF7 (b) and T47D (c) cells transfected with GATA3 siRNA relative to control transfected cells, both normalized to beta-actin. Results are average ± standard error of three to five independent experiments. *T-Test P-values <0.1; **T-Test P-values <0.05; ***T-Test P-values <0.01. (PDF 272 KB) [file 13058_2014_464_MOESM4_ESM.pdf]
