# Supplementary figures and images for: Shift in GATA3 functions, and GATA3 mutations, control progression and clinical presentation in breast cancer
Source: Breast Cancer Res. 2014 Nov 20;16:464. doi: 10.1186/s13058-014-0464-0 (PMC4303202; doi:10.1186/s13058-014-0464-0)

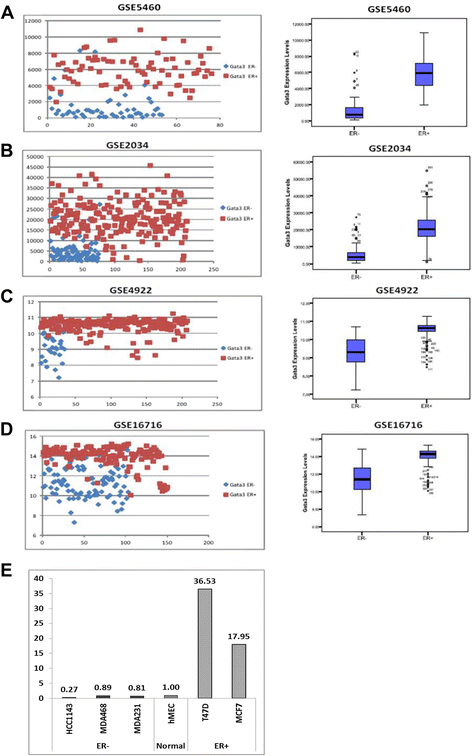

Supplement: Supplementary file 5 — Authors’ original file for figure 1 [file 13058_2014_464_MOESM5_ESM.gif]

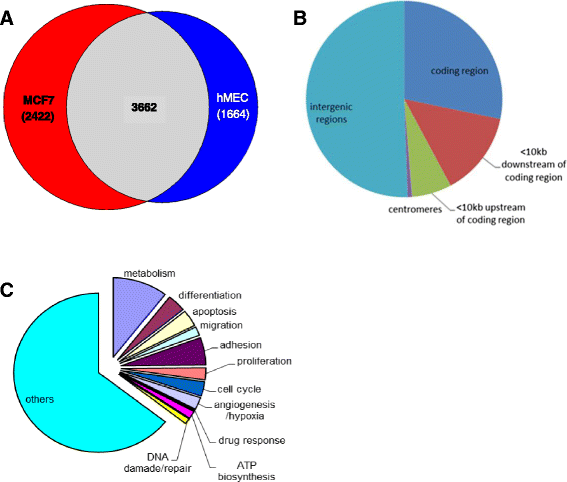

Supplement: Supplementary file 6 — Authors’ original file for figure 2 [file 13058_2014_464_MOESM6_ESM.gif]

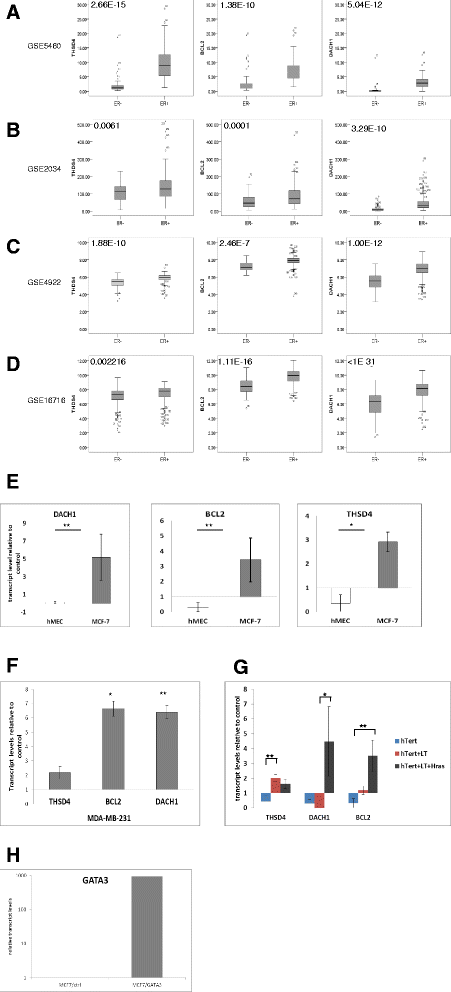

Supplement: Supplementary file 7 — Authors’ original file for figure 3 [file 13058_2014_464_MOESM7_ESM.gif]

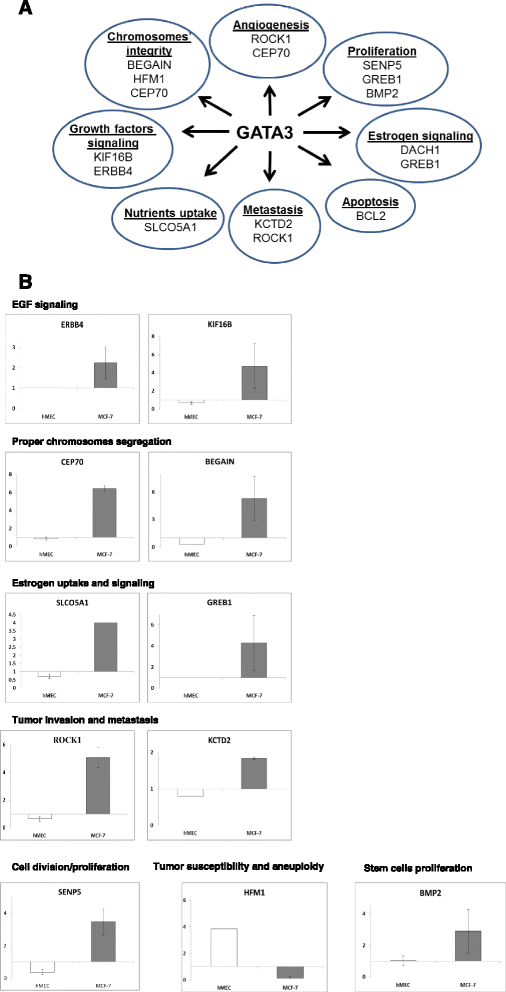

Supplement: Supplementary file 8 — Authors’ original file for figure 4 [file 13058_2014_464_MOESM8_ESM.gif]

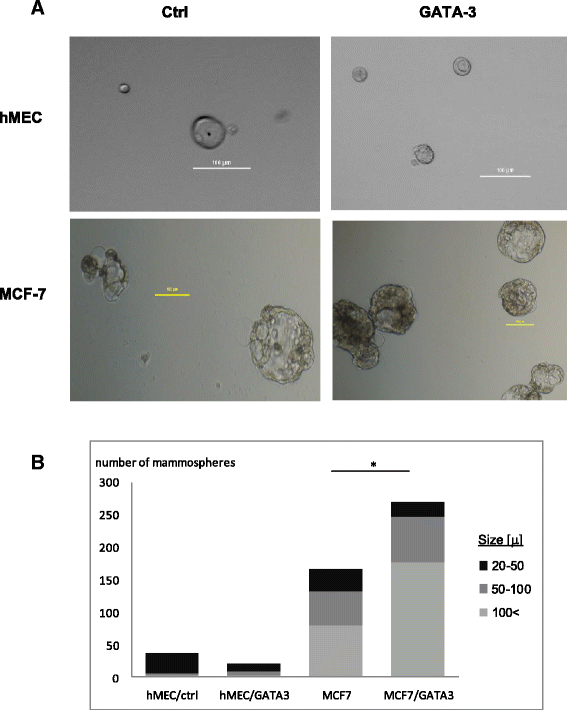

Supplement: Supplementary file 9 — Authors’ original file for figure 5 [file 13058_2014_464_MOESM9_ESM.gif]

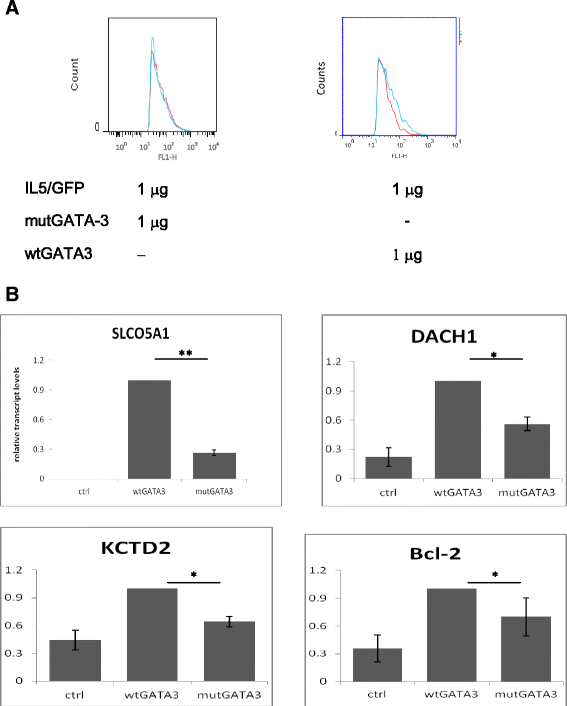

Supplement: Supplementary file 10 — Authors’ original file for figure 6 [file 13058_2014_464_MOESM10_ESM.gif]

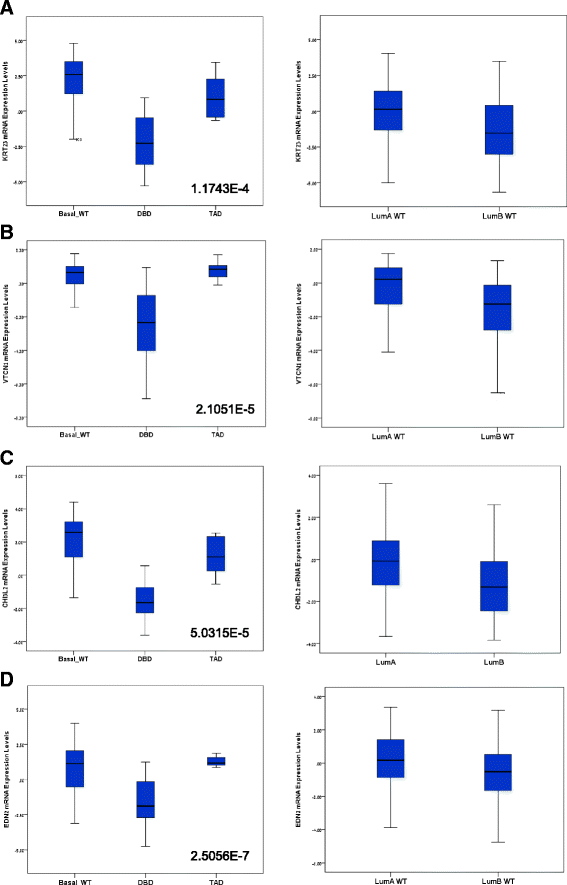

Supplement: Supplementary file 11 — Authors’ original file for figure 7 [file 13058_2014_464_MOESM11_ESM.gif]
